# Microbiota–Inflammation Crosstalk in Myeloproliferative Neoplasms: MPN-Specific Human Data, Mechanistic Plausibility and Translational Priorities

**DOI:** 10.3390/biomedicines14092074

**Published:** 2026-09-15

**Authors:** Laura-Gabriela Țîrlea, Lavinia Lipan, Alina Daniela Tănase

**Affiliations:** 1 Doctoral School Department, University of Medicine and Pharmacy “Carol Davila”, 020021 Bucharest, Romania; lavinia.lipan@icfundeni.ro; 2 Colentina Clinical Hospital, Hematology I, 020125 Bucharest, Romania; 3 Bone Marrow Transplant Unit, Fundeni Clinical Institute, 022328 Bucharest, Romania

**Keywords:** myeloproliferative neoplasms, gut microbiota, dysbiosis, *JAK2V617F*, inflammation, gut–bone marrow axis, metabolome, thrombo-inflammation, diet, fecal microbiota transplantation, multi-omics

## Abstract

Myeloproliferative neoplasms (MPNs) are clonal hematopoietic stem cell disorders driven mainly by somatic mutations in *JAK2*, *CALR* or *MPL*, but their clinical phenotype is also shaped by chronic inflammation, immune dysregulation, vascular complications and microenvironmental remodeling. Emerging evidence suggests that the gut microbiota may contribute to this inflammatory and immunometabolic landscape; however, the current literature remains heterogeneous and its translational relevance is still insufficiently defined. This critical narrative review maps the available evidence linking the gut microbiota, microbial metabolites and systemic microbial signatures to MPN biology. We distinguish direct human MPN data from indirect mechanistic evidence derived from studies of intestinal barrier dysfunction, thrombo-inflammation, hematopoietic regulation, allogeneic hematopoietic cell transplantation and infection risk. Across human MPN cohorts, the most consistent findings are not uniform changes in global microbial diversity, but rather alterations in specific immunoregulatory taxa, particularly reduced *Firmicutes*/*Faecalibacterium*-related communities and dysbiotic signatures associated with *JAK2V617F* status. Mechanistically, dysbiosis and impaired intestinal barrier integrity may facilitate low-grade endotoxemia, TLR4/NF-κB activation, cytokine amplification, endothelial activation and platelet priming. In parallel, microbial metabolites may influence hematopoietic stem cell programs, the bone marrow niche, megakaryopoiesis and thrombopoiesis. Treatment exposure and diet are relevant modifiers of the microbiota–inflammation axis, although available interventional data remain preliminary. Mendelian randomization and multi-omics studies provide hypothesis-generating evidence for microbiota–metabolome–MPN interactions, but require longitudinal validation, functional studies and contamination-aware analytical pipelines, especially for low-biomass blood and bone marrow samples. Microbiota-targeted strategies, including nutritional interventions and fecal or washed microbiota transplantation, represent promising but still investigational approaches, particularly in immunocompromised or post-transplant settings. Future studies should integrate microbiome, metabolome, genome, proteome, inflammatory biomarkers and clinical outcomes while controlling for diet, antibiotics, treatment exposure and driver mutation status. Such an approach may clarify whether the microbiota is a biomarker, mediator or therapeutic target in MPNs.

## 1. Introduction

Myeloproliferative neoplasms (MPNs) are clonal hematopoietic stem cell disorders that include polycythemia vera (PV), essential thrombocythemia (ET), primary myelofibrosis (PMF) and MPN-unclassifiable (MPN-U). Their molecular pathogenesis is largely driven by somatic mutations affecting the JAK–STAT pathway, most frequently *JAK2V617F*, *CALR* and *MPL*, although additional molecular lesions and host-related factors contribute to phenotypic heterogeneity, clonal evolution and disease progression [1,2].

Beyond clonal hematopoiesis, MPNs are characterized by a sustained inflammatory state that contributes to constitutional symptoms, vascular complications, thrombosis, bone marrow remodeling and disease evolution. Inflammation may arise both from the malignant clone and from non-mutated immune and stromal cells, creating a self-amplifying network of cytokine production, innate immune activation, endothelial dysfunction and altered hematopoietic regulation [3,4,5]. This inflammatory dimension has shifted the conceptual understanding of MPNs from purely mutation-driven disorders toward biologically complex diseases in which genetic, immune, metabolic and microenvironmental factors interact.

The gut microbiota has emerged as a potential modulator of systemic inflammation, immune homeostasis and hematopoiesis. Dysbiosis, defined as an alteration in microbial composition or function, is not necessarily pathogenic by itself, but may acquire biological relevance when associated with impaired intestinal barrier integrity, altered microbial metabolite production or systemic translocation of microbial products [6,7,8]. Microbiota-derived metabolites, including short-chain fatty acids, secondary bile acids, indoles, polyamines and lipid derivatives, can influence innate and adaptive immunity through receptor-mediated, metabolic and epigenetic mechanisms [7]. In parallel, microbial products such as lipopolysaccharides may promote low-grade endotoxemia, TLR4 signaling, NF-κB activation and thrombo-inflammatory responses, providing a biologically plausible bridge between intestinal dysbiosis, systemic inflammation and vascular risk [8,9].

The relevance of the gut–bone marrow axis has also become increasingly apparent. Experimental and translational studies suggest that microbial signals can influence hematopoietic stem cell fate, bone marrow niche function, stromal cell activity, inflammatory signaling and lineage differentiation [10]. These mechanisms are particularly relevant in MPNs, where chronic inflammation, megakaryocytic proliferation, myeloid skewing and marrow remodeling are central biological features. However, much of the mechanistic evidence connecting microbiota-derived signals to hematopoiesis comes from preclinical models or non-MPN contexts and should therefore be interpreted as mechanistic plausibility rather than direct proof of causality in patients with MPNs.

Human data specifically addressing the gut microbiota in MPNs remain limited but increasingly informative. Available fecal microbiome studies suggest that MPN patients have subtle microbial alterations, particularly involving reduced immunoregulatory taxa such as *Faecalibacterium* and other Firmicutes-related communities [11,12,13]. Importantly, emerging data indicate that *JAK2V617F* status may be associated with more pronounced differences in gut microbial composition, whereas *CALR*-mutated patients may show microbiota profiles closer to those of healthy controls [13]. Other studies suggest that treatment exposure, particularly interferon-based therapy, and nutritional interventions are relevant modifiers of the microbiota–inflammation axis, although the magnitude, durability and clinical relevance of these changes remain uncertain [14,15].

At the same time, newer methodological approaches have expanded the field beyond classical stool-based microbiome profiling. Mendelian randomization and mediation analyses have suggested potential causal relationships between gut microbial taxa, plasma metabolites and MPN risk, but these findings remain hypothesis generating and require longitudinal and functional validation [16]. Multi-omics studies of circulating microbial content or bone marrow-derived microbial molecular signals may offer diagnostic and prognostic opportunities, but they also raise important methodological concerns, particularly because blood and bone marrow are low-microbial-biomass compartments that are highly vulnerable to contamination and batch effects [17,18,19].

Although several reviews have discussed inflammation, microenvironmental regulation or microbiota in hematologic malignancies, the specific role of the gut microbiota in MPNs remains insufficiently integrated into a critical evidence-based framework. Available studies differ substantially in design, population, sequencing method, biological sample type, treatment exposure, dietary assessment, antibiotic exposure and clinical endpoints. Moreover, direct human MPN data are often discussed together with indirect evidence from cardiovascular inflammation, intestinal barrier biology, hematopoietic regulation or allogeneic transplantation, which may obscure the actual strength of the evidence.

Therefore, the aim of this review is not only to summarize the emerging literature on microbiota in MPNs, but to critically evaluate the strengths, limitations and translational relevance of the current evidence. We propose a gut–bone marrow axis model linking dysbiosis, intestinal barrier dysfunction, microbial metabolites, chronic inflammation, driver mutation status and clinical phenotype. In doing so, we distinguish established observations from mechanistic plausibility and hypothesis-generating data, and we identify the main research priorities required before microbiota-based biomarkers or interventions can be incorporated into MPN care.

## 2. Materials and Methods

This article was designed as a critical narrative review with structured evidence categorization, aiming to synthesize and contextualize the available literature on the relationship between gut microbiota, inflammation and myeloproliferative neoplasms (MPNs). The objective was not to perform a systematic review or formal evidence map, but to critically distinguish findings derived from MPN-specific human studies from mechanistic, causal inference, multi-omics, transplantation-related and supportive-care evidence derived from broader or adjacent contexts.

The PubMed literature search was initially conducted during manuscript preparation and was updated on 28 August 2026 to capture publications newly published or indexed since the previous search performed in June 2026. The updated MPN-focused search identified 37 records using predefined combinations of MPN-related and microbiome-related terms. The complete search strategy is provided in Supplementary Table S1.

The literature search, title and abstract screening, full-text assessment, study selection and initial evidence categorization were performed by the first author. The resulting synthesis and interpretation of the evidence were subsequently reviewed and critically revised by all authors. Given the narrative design of the review, duplicate independent screening and formal adjudication of disagreements were not performed.

Records were screened at the title and abstract level, followed by full-text assessment of potentially relevant publications. Publications were retained when they addressed gut microbiota composition, microbial molecular signatures, microbial metabolites linked to microbial biology, microbiota-directed interventions, or microbiota-related biological mechanisms in patients with MPNs or in directly relevant MPN models. Records were excluded when the microbiome component was absent or incidental, when they focused on unrelated diseases or non-MPN forms of erythrocytosis or myeloid disease, or when metabolomic or inflammatory findings were not linked to microbial biology.

Because the scope of this critical narrative review extends beyond MPN-specific clinical studies, additional targeted PubMed searches were used to identify the mechanistic, methodological and translational studies required to interpret the disease-specific findings. These searches addressed intestinal barrier dysfunction, microbial metabolites, gut–immune interactions, lipopolysaccharide/Toll-like receptor signaling, thrombo-inflammation, hematopoietic regulation, the bone marrow microenvironment, low-microbial-biomass methodology, allogeneic hematopoietic cell transplantation, graft-versus-host disease, microbiota transplantation and infectious safety. Recent contextual reviews addressing systemic microbiota–immune interactions and strain-specific immunomodulatory mechanisms were also considered when they provided relevant mechanistic or translational context. Consequently, the final reference set includes both publications retrieved through the updated MPN-focused search and contextual sources identified through targeted searches. Articles published from 2017 to August 2026 were preferentially considered, while earlier publications were retained when they provided essential mechanistic, methodological or safety-related context.

Eligible evidence types included observational human studies, nutritional and other microbiota-related intervention studies, preclinical models, Mendelian randomization analyses, multi-omics studies, case reports, case series, safety reports, methodological studies and relevant reviews. Publications were excluded when they did not address microbiota-related biology, did not include MPNs or a clearly relevant hematologic or transplantation context, or provided only general microbiome information without mechanistic, methodological or translational relevance to the topic.

For the structured synthesis, publications were designated as key evidence sources when they provided primary MPN-specific data or substantive mechanistic, causal inference, multi-omics, translational, safety or methodological findings that were directly discussed in the review. References used primarily to support disease classification, general MPN biology, microbiota definitions or broader methodological background were retained in the narrative synthesis but were not classified as key evidence sources.

Evidence was organized into five interpretative domains: MPN-specific human studies, mechanistic evidence derived primarily from preclinical or non-MPN settings, exploratory causal inference and multi-omics studies, adjacent hematologic and transplantation-related evidence, and supportive-care or intervention-related evidence. These categories describe the clinical or biological context and directness of the evidence and do not represent a formal hierarchy of methodological quality or certainty. Accordingly, disease-specific relevance was not interpreted as equivalent to high-certainty evidence.

For each key evidence source, the study design, population or model, biological sample type, microbiome-related methodology, principal findings, relevance to MPN biology and major limitations were considered. Particular attention was given to potential confounders, including MPN subtype, driver mutation status, treatment exposure, antibiotic use, diet, sample type, sequencing methodology and the distinction between fecal, circulating and bone marrow-derived microbial signals. Findings from low-microbial-biomass compartments, particularly blood and bone marrow, were interpreted cautiously because of the recognized risks of contamination, batch effects and analytical variability.

The synthesis was structured to distinguish MPN-specific observations from mechanistic plausibility and hypothesis-generating findings. Mendelian randomization and mediation analyses were interpreted as exploratory causal inference approaches rather than definitive demonstrations of biological causality. Similarly, microbiota-directed interventions, including nutritional strategies and fecal or washed microbiota transplantation, were considered investigational unless supported by prospective clinical evidence and appropriate safety monitoring.

As this was a critical narrative review, no quantitative meta-analysis, formal risk-of-bias scoring or PRISMA-based systematic selection process was undertaken. The structured search and evidence categorization were intended to improve transparency and reproducibility while preserving the narrative and hypothesis-generating nature of the review. The evidence categorization framework is summarized in Table 1.

## 3. Biological Definitions and Conceptual Framework

The term gut microbiota refers to the complex ecological community of microorganisms inhabiting the gastrointestinal tract and contributing to host metabolic, immune and barrier homeostasis. In this context, dysbiosis should be understood as an alteration in microbial composition, diversity or function, rather than as an intrinsically pathogenic state. Importantly, taxonomic differences alone do not necessarily indicate functional dysbiosis; demonstrating functional alteration requires complementary assessment of microbial genes, pathways, metabolites or host–microbial interactions. Its biological relevance depends on the functional consequences of microbial imbalance, including changes in metabolite production, impaired colonization resistance, mucosal immune activation and increased intestinal permeability [6,7].

Microbiota-derived metabolites represent key mediators of host–microbe communication. Short-chain fatty acids, secondary bile acids, indole derivatives, polyamines and lipid mediators can influence innate and adaptive immunity through receptor-mediated signaling, metabolic regulation and epigenetic mechanisms [7]. These metabolites may affect immune-cell differentiation, epithelial barrier integrity and inflammatory tone, with effects that vary according to concentration, anatomical compartment and host context. This context dependency is particularly important when interpreting microbiota-related findings in chronic inflammatory diseases such as MPNs.

The intestinal barrier provides a critical interface between the luminal microbial ecosystem and the systemic immune compartment. It is maintained through coordinated biological, immune and mechanical components. The biological barrier is represented by commensal microorganisms that contribute to colonization resistance and metabolic homeostasis. The immune barrier includes gut-associated lymphoid tissue, secretory IgA, regulatory and effector T cells, innate lymphoid cells, macrophages and dendritic cells. The mechanical barrier is formed mainly by the epithelial layer and intercellular tight junctions, which restrict paracellular passage of microbial products and inflammatory mediators [20].

Studying the gut microbiota in MPNs is biologically relevant because the intestinal ecosystem may interact with inflammatory, immune, vascular and hematopoietic processes that are also central to MPN biology [3,4,5,10]. However, most proposed links involving intestinal barrier dysfunction, microbial translocation, microbial metabolites, endothelial or platelet activation and hematopoietic regulation are derived from preclinical or non-MPN settings [7,8,9,10,20,21]. In the context of MPNs, these pathways should therefore be considered hypothesis-generating mechanisms rather than established disease-specific processes.

MPN-specific human studies have reported differences in gut microbial composition in association with inflammatory phenotype, driver mutation status and treatment exposure [11,12,13,14]. Additional studies in PV have described circulating extracellular-vesicle-associated microbial DNA signatures [22,23]. These observations remain predominantly associative, and their functional consequences, directionality and independence from clinical confounders have not been established. Accordingly, no microbiota-based biomarker or microbial profile is currently validated for diagnosis, risk stratification or disease monitoring in MPNs. The conceptual framework used in this review therefore distinguishes MPN-specific observations from indirect mechanistic plausibility and provides the basis for the gut–bone marrow working model discussed below.

## 4. Hypothesis-Generating Mechanistic Links Between Gut Microbiota and MPN Biology

Several non-mutually exclusive mechanisms may provide biological plausibility for interactions between the intestinal microbiota and MPN biology, including intestinal barrier dysfunction, microbial translocation, innate immune and thrombo-inflammatory signaling, microbiota-derived metabolites and gut–bone marrow crosstalk. Except where explicitly stated, the evidence supporting these pathways is derived predominantly from preclinical studies or non-MPN inflammatory, cardiovascular and hematopoietic settings. Accordingly, the mechanisms discussed below should be regarded as hypothesis generating rather than as established MPN-specific pathways.

### 4.1. Intestinal Barrier Dysfunction and Low-Grade Endotoxemia

Experimental studies indicate that microbial products such as lipopolysaccharides (LPSs) can impair intestinal barrier integrity and increase paracellular permeability through inflammatory signaling and tight-junction dysregulation [8,20]. Increased intestinal permeability may facilitate systemic exposure to microbial products and thereby contribute to low-grade innate immune activation.

This mechanism has not been directly demonstrated in MPN cohorts. Nevertheless, because chronic inflammation and cytokine dysregulation are recognized features of MPN biology [3,4], impaired barrier function and microbial translocation represent biologically plausible modifiers of systemic inflammation rather than established components of MPN pathogenesis.

### 4.2. Innate Immune Activation and Thrombo-Inflammation

Systemic exposure to microbial products such as LPS may promote thrombo-inflammatory signaling through activation of Toll-like receptor 4 (TLR4)-dependent pathways in monocytes, endothelial cells, neutrophils and platelets, with downstream effects on cytokine release, endothelial activation and procoagulant responses [9]. These mechanisms are well described in cardiovascular and inflammatory settings and provide biological plausibility for a potential microbiota-related contribution to thrombo-inflammation.

This concept is relevant to MPNs because inflammation, endothelial dysfunction, leukocyte–platelet interactions and coagulation activation are recognized contributors to the thrombotic phenotype of these disorders [3,4,5]. However, a microbiota–LPS–thrombo-inflammation axis has not been directly demonstrated in MPN cohorts. Accordingly, low-grade endotoxemia should be regarded as a potential modifier of thrombo-inflammatory signaling rather than an established driver of MPN-associated thrombosis.

### 4.3. Microbiota-Derived Metabolites and Immune Regulation

Microbiota-derived metabolites, including short-chain fatty acids, secondary bile acids and indole derivatives, represent important mediators of host–microbiota communication and can influence epithelial barrier integrity, immune-cell function and systemic inflammatory tone through receptor-mediated, metabolic and epigenetic mechanisms [7]. Their biological effects are context-dependent and may vary according to metabolite concentration, tissue compartment and host inflammatory state.

In MPNs, microbiota-derived metabolites could theoretically interact with cytokine signaling, immune regulation and hematopoietic or bone marrow microenvironmental responses [3,4,10,21]. However, direct functional evidence for these pathways in MPN patients remains limited. An MPN-focused Mendelian randomization and mediation analysis has suggested potential relationships among gut microbial features, plasma metabolites and MPN risk [16], but such findings should be regarded as exploratory causal inference evidence rather than direct demonstration of a microbiota–metabolite mechanism in MPNs. Future studies should integrate longitudinal stool microbiome profiling with plasma metabolomics, inflammatory biomarkers, molecular disease characteristics and clinical outcomes.

### 4.4. A Bidirectional Gut–Bone Marrow Working Model

The gut–bone marrow axis provides a conceptual framework for integrating microbial, inflammatory and hematopoietic signals. Experimental and translational evidence indicates that gut-derived microbial products and metabolites can influence systemic inflammatory and metabolic cues involved in the regulation of hematopoietic stem and progenitor cells, stromal cell function and lineage differentiation [10,21]. Microbiota-dependent effects on megakaryopoiesis and platelet production have also been described experimentally, although their direction and magnitude appear to depend on the biological context [21].

**Figure 1:**
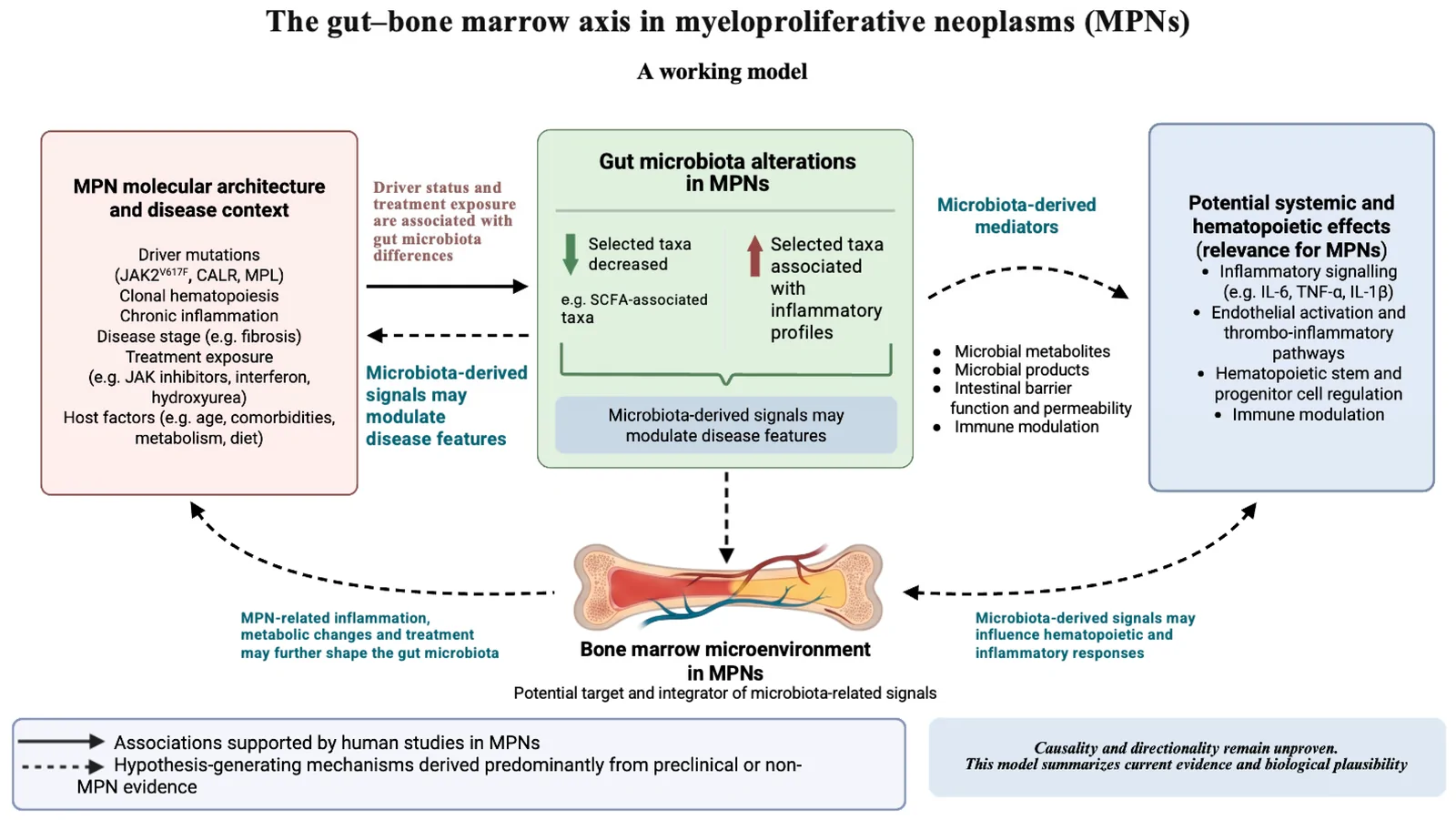
Proposed bidirectional gut–bone marrow axis in myeloproliferative neoplasms: a working model. MPN-specific human studies support associations between driver mutation status, treatment exposure and gut microbial composition, whereas the proposed effects of microbiota-derived metabolites, microbial products and barrier-related signals on systemic inflammation and hematopoietic responses remain predominantly hypothesis generating [7,8,9,10,11,12,13,14,21,24,25]. Solid arrows indicate associations supported by MPN-specific human studies; dashed arrows indicate mechanisms derived mainly from preclinical or non-MPN evidence. Causality and directionality remain unproven. Created in BioRender. Tirlea, L. (2026) https://BioRender.com/1fwye4m.

In MPNs, this framework is particularly relevant because clonal hematopoiesis, chronic inflammation, altered megakaryopoiesis and bone marrow remodeling coexist within the same disease process [1,2,3,4]. A bidirectional working hypothesis can therefore be proposed: the molecular and inflammatory architecture of MPNs may influence the intestinal microbial ecosystem through systemic inflammation, treatment exposure and host-related metabolic changes, while microbiota-derived products and metabolites may, in turn, modify inflammatory tone and hematopoietic or stromal responses. MPN-specific human studies reporting associations between gut microbial composition and JAK2V617F status provide preliminary support for an interaction between disease molecular architecture and the intestinal ecosystem [12,13]. However, these studies do not establish the direction or causality of this relationship. These interactions are summarized conceptually in Figure 1, which distinguishes MPN-specific human associations from mechanistic pathways that remain predominantly hypothesis generating.

Accordingly, the proposed gut–bone marrow axis should be regarded as a working model rather than a demonstrated pathogenic pathway in MPNs. A central unresolved question is whether microbiota alterations primarily represent a consequence of disease biology and treatment exposure, a modifier of inflammatory phenotype, or a correlated biomarker. Longitudinal studies integrating microbiome profiling, microbial and host metabolomics, inflammatory biomarkers, driver mutation status and allele burden, treatment exposure and clinical phenotype will be required to distinguish among these possibilities.

## 5. MPN-Specific Human Studies: Microbial Signatures, Driver Mutations, Treatment Exposure and Diet

MPN-specific human data on intestinal microbiota and microbiota-related molecular signals remain limited. The available evidence includes small observational cohorts, an early conference abstract, a randomized dietary pilot study and exploratory analyses of circulating microbial DNA. Collectively, these studies report taxonomic differences and microbiota-related molecular signals associated with MPN phenotype, inflammatory status, driver mutation status, treatment exposure and dietary context, but they do not define a uniform or functionally validated MPN-associated dysbiosis profile [11,12,13,14,15,22,23,26,27]. Interpretation is further complicated by potential confounders, including age, body mass index, diet, antibiotic exposure, treatment, infections and metabolic comorbidities, which have not been uniformly assessed or controlled across studies.

An early MPN-specific report was presented as a *Blood* conference abstract in 2018 [26]. Patients with ET, PV and MF were reported to have increased inflammatory cytokines together with a higher abundance of Prevotellaceae, while microbial composition also appeared to differ according to treatment exposure, including ruxolitinib and hydroxyurea [26]. Because the report was available only as a conference abstract, with limited methodological information, its findings should be considered hypothesis generating. Moreover, several investigators subsequently contributed to a more detailed study by Oliver et al. [11]; therefore, the 2018 abstract should not be assumed to represent an independent replication, and the extent of participant overlap cannot be established from the available report.

Oliver et al. subsequently analyzed triplicate fecal samples from 25 patients with MPNs and 25 non-MPN controls using 16S rRNA gene sequencing [11]. Most variation in microbial community composition was attributable to interindividual differences, whereas MPN status accounted for only 1.7% of the observed variance. Nevertheless, taxon-specific differences were identified, including lower abundance of Phascolarctobacterium in patients with MPNs, together with associations between selected bacterial taxa and inflammatory cytokines [11]. These findings support an association between selected microbial features and the inflammatory phenotype of MPNs, while also illustrating that disease-associated differences are modest relative to the substantial interindividual variability of the gut microbiota.

The relationship between MPN molecular characteristics and gut microbiota composition has been investigated most extensively within a Danish research program [12,13,14]. In a study of 54 patients with ET and 42 healthy controls, Eickhardt-Dalbøge et al. reported differences in microbial richness and composition, together with a lower abundance of several taxa with proposed immunoregulatory properties; microbial differences were more pronounced in JAK2V617F-positive than in JAK2V617F-negative ET [12]. In a PV cohort comprising 102 patients and 42 healthy controls, microbial composition differed from that of controls and also varied across treatment groups [14]. A subsequent expanded analysis included 227 patients with ET, PV, prefibrotic PMF or overt MF and 42 healthy controls, and reported lower abundance of several Firmicutes-related taxa, including Faecalibacterium, together with stronger associations of microbial composition with JAK2V617F status than with clinical MPN subtype alone [13]. CALR-mutated patients showed microbial profiles more similar to healthy controls than did JAK2V617F-positive patients [13].

Importantly, these publications do not represent three independent replications. The expanded 2024 study explicitly incorporated the investigators’ previous PV and ET datasets and added patients with PV, prefibrotic PMF and overt MF [13]. Accordingly, the association between JAK2V617F status and gut microbial composition is a repeatedly observed finding within an expanded research cohort, but its independent external replication remains limited [12,13,14].

Treatment exposure represents an important potential modifier and confounder of these associations. In the PV study, gut microbial composition differed among patients receiving interferon-α, hydroxyurea, no cytoreductive therapy or combination therapy, and interferon-treated patients were reported to have microbial profiles closer to those of healthy controls [14]. However, the cross-sectional design cannot determine whether these differences reflect a direct treatment effect, underlying differences in disease biology or treatment selection, or other unmeasured clinical and environmental factors. The available evidence therefore does not establish that interferon or other MPN-directed therapies remodel the gut microbiota. Longitudinal sampling before and after treatment initiation will be required to address this question [14].

Dietary modulation has been evaluated prospectively in the NUTRIENT trial [15,27]. Thirty-one patients with MPNs were randomized to Mediterranean-style or standard U.S. dietary counseling, of whom 28 completed the 15-week study and constituted the principal analyzed cohort [15,27]. Detailed shotgun metagenomic analysis showed that overall microbiome diversity and composition remained stable during the intervention, although associations were observed between microbial features, MPN characteristics and inflammatory cytokines [27]. The clinical trial report demonstrated that Mediterranean-style dietary counseling was feasible and well tolerated, with improvement in symptom burden observed in both dietary groups; however, no significant changes in inflammatory cytokines or overall gut microbiome diversity and composition were demonstrated over the short intervention period [15].

References [15,27] represent complementary reports from the same NUTRIENT trial rather than independent intervention cohorts. Their findings support the feasibility of dietary intervention and provide an MPN-specific framework for studying diet–microbiota–inflammation relationships, but they do not demonstrate a microbiota-modifying, anti-inflammatory or disease-modifying effect of Mediterranean-style dietary counseling in MPNs [15,27].

Beyond stool-based profiling, two related studies from the same investigative group evaluated plasma-derived extracellular vesicles (EVs) and EV-associated microbial DNA cargo in PV [22,23]. One study analyzed 38 patients with PV and 30 healthy donors and reported differences in circulating EV phenotypes together with greater diversity and altered composition in EV-associated microbial DNA in PV; in contrast, fecal microbiome profiling did not clearly distinguish patients with PV from healthy donors [22]. A second analysis involving 38 patients with PV identified EV-associated host and microbial DNA patterns associated with previous thrombosis and marrow fibrosis [23]. Because both reports derive from the same investigative program, involve the same-sized PV population and use closely related EV-based approaches, they should be considered related evidence rather than independent validation unless participant independence can be established.

These circulating microbial-DNA findings should also be distinguished from evidence of a circulating or tissue-resident microbiome. Blood-derived microbial analyses involve low-microbial-biomass material and are particularly vulnerable to contamination, reagent-derived signals, batch effects and analytical variability [19]. Accordingly, the findings in PV should be interpreted as candidate molecular signatures requiring independent technical and clinical validation rather than as evidence of a validated microbiota-based biomarker [19,22,23].

Taken together, MPN-specific human studies indicate that gut microbial composition and microbiota-related molecular signals have been reported in association with inflammatory phenotypes, driver mutation status, treatment exposure and dietary context [11,12,13,14,15,22,23,26,27]. The association between JAK2V617F-positive disease and altered gut microbial composition is the most consistently reported disease-specific signal to date, particularly within the Danish datasets [12,13,14]. However, these observations arise partly from overlapping patient populations, and independent external replication remains limited. Taxonomic differences alone do not establish functionally relevant dysbiosis, and the direction of the observed relationships remains unresolved: microbiota alterations may reflect a consequence of MPN biology or treatment exposure, act as modifiers of the inflammatory phenotype, or represent correlated disease markers. At present, no microbiota-based biomarker or microbial profile has been validated for MPN diagnosis, risk stratification or treatment monitoring, and the available evidence is insufficient to recommend microbiota-targeted interventions as part of standard MPN therapy.

## 6. Exploratory Preclinical, Causal Inference and Multi-Omics Evidence

Beyond MPN-specific human studies, preclinical models, Mendelian randomization analyses and multi-omics studies provide complementary hypothesis-generating evidence. These approaches can help identify candidate biological mechanisms and molecular signals, but they do not represent a linear progression from association to demonstrated causality. Their relevance to MPN biology varies according to study design, population, biological compartment and analytical assumptions [16,17,18,19,24,25,28,29,30].

### 6.1. Preclinical Models Supporting Biological Plausibility

Two preclinical models provide biological plausibility for interactions among intestinal microbiota, environmental exposures and hematopoiesis. Tadokoro et al. showed that, in Spred1-deficient mice, high-fat diet exposure induced ERK hyperactivation, abnormal hematopoietic stem cell self-renewal, severe anemia and an MPN-like disease phenotype; importantly, the hematopoietic abnormalities were partly mediated by alterations in gut microbiota [24]. Josefsdottir et al. demonstrated that broad-spectrum antibiotic-induced depletion of intestinal microbiota in mice was associated with anemia, thrombocytosis, leukopenia and depletion of hematopoietic stem and multipotent progenitor cells, with partial correction of hematopoietic abnormalities after fecal microbiota transfer [25]. Germ-free mice showed similar hematopoietic abnormalities, further supporting a role of the intestinal microbiota in maintaining hematopoietic homeostasis [25].

These experimental findings demonstrate that perturbation of the intestinal microbiota can modify hematopoietic homeostasis in murine systems, but neither model reproduces classical human MPN biology. They should therefore be interpreted as preclinical evidence of biological plausibility rather than as evidence that microbiota alterations initiate or drive MPNs in patients [24,25].

### 6.2. Exploratory Mendelian Randomization and Microbiota–Metabolome Analyses

Mendelian randomization (MR) has been applied to explore genetically proxied relationships between gut microbial traits, circulating metabolites and MPN risk [16,28]. Kan et al. performed a two-sample MR analysis integrating genetic instruments for 196 gut microbial taxa and 526 plasma metabolites with MPN outcome data, together with mediation and multivariable MR analyses [16]. The study reported MR associations involving seven microbial taxa and seventeen plasma metabolites. In subsequent mediation analyses, the Eubacterium xylanophilum group was linked to MPN risk through models involving succinylcarnitine and lysine [16]. These findings identify candidate microbiota–metabolite relationships for further investigation but do not demonstrate that these taxa or metabolites biologically cause MPNs.

A broader bidirectional MR study by Chen et al. evaluated genetically predicted gut microbial traits in relation to several hematologic malignancies [28]. Within the MPN analysis, the authors reported associations involving Coprococcus 3 and the Eubacterium hallii group [28]. Because this study evaluated multiple hematologic malignancies using genome-wide association study (GWAS)-based microbial traits rather than microbiota measured directly in patients with MPNs, its findings provide exploratory causal inference evidence rather than disease-specific functional validation.

The interpretation of microbiome-related MR requires particular caution. MR estimates depend on the validity of genetic instruments and on assumptions concerning instrument relevance, independence from confounding and the absence of pathways from the instrument to the outcome other than through the exposure of interest [30]. In microbiome applications, interpretation is further complicated by limited taxonomic resolution, the population dependence of microbial GWAS datasets, substantial interindividual variability, potential horizontal pleiotropy and the uncertain correspondence between genetically predicted microbial abundance and clinically modifiable microbial exposure [16,28,30]. Accordingly, MR findings in MPNs should be regarded as hypothesis-generating evidence that prioritizes candidate taxa and metabolites for replication and functional testing rather than as proof of microbiota-mediated causality.

### 6.3. Multi-Omics Studies and Candidate Microbial Molecular Signals

Multi-omics and sequencing-based approaches have extended microbiota-related research beyond fecal profiling by examining microbial molecular signals in blood and bone marrow-derived samples [17,18]. These studies may reveal associations between microbial DNA-derived features, host molecular characteristics and disease phenotype, but their interpretation requires particular caution because blood and bone marrow are low-microbial-biomass compartments in which contamination, reagent-derived signals and batch effects can substantially influence results [19].

Woerner et al. analyzed existing deep DNA-sequencing data from blood or bone marrow samples from 1870 patients with myeloid malignancies, including 354 patients with MPNs, and evaluated bacterial, fungal and viral sequence content [17]. Microbial molecular patterns differed across myeloid disease subtypes and were associated with several host and disease characteristics, including somatic mutations and blast percentage. Machine learning models based on bacterial content showed some ability to distinguish MPNs from other myeloid malignancies, while classification of JAK2-mutated versus JAK2-wild-type MPNs showed more limited discriminatory performance [17]. Importantly, the study did not demonstrate a prognostic microbial signature in the MPN subgroup; the reported association between Epstein–Barr virus detection and overall survival was confined to patients with myelodysplastic syndrome. Thus, the MPN-related findings primarily support associations between circulating microbial DNA-derived signals, disease classification and molecular features rather than validated prognostic utility.

An MPN-specific multi-omics study by Zhang et al. evaluated formalin-fixed, paraffin-embedded bone marrow biopsies from 13 untreated JAK2V617F-positive patients with ET or prefibrotic PMF using proteomic profiling and 2bRAD-M sequencing [18]. The investigators reported differences in protein expression and microbial DNA-derived features between the two disease groups, including inflammatory, immune and lipid-related proteomic pathways and differential abundance of several bacterial genera [18]. Some of these features showed discriminatory performance within the study cohort. However, the sample comprised only seven patients with ET and six with prefibrotic PMF, and no independent validation cohort was included. Given the low-biomass nature of bone marrow material, these findings should be considered exploratory molecular associations rather than evidence of a distinct bone marrow microbiome or clinically validated diagnostic markers [18,19].

Overall, these studies illustrate the potential of integrated molecular approaches to generate candidate microbial and host-associated signals for further investigation [17,18]. At present, however, neither circulating nor bone marrow-derived microbial molecular signatures have demonstrated sufficient independent reproducibility, biological validation or clinical performance to support diagnostic, prognostic or risk-stratification use in MPNs.

## 7. Adjacent Clinical Context: Allo-HCT, Infection Risk and Microbiota-Directed Interventions in Advanced MPNs

Microbiota-related evidence from allogeneic hematopoietic cell transplantation (allo-HCT) provides an adjacent clinical context rather than direct evidence for microbiota involvement in classical MPN pathogenesis. Allo-HCT is associated with substantial disruption of the intestinal microbiota through conditioning therapy, antimicrobial exposure, dietary changes and mucosal injury, and reduced microbial diversity and intestinal domination have been associated with graft-versus-host disease (GVHD), infections and adverse transplant outcomes [31]. These observations are most relevant to patients with myelofibrosis or other advanced MPNs who undergo allo-HCT and should not be extrapolated to the broader non-transplant MPN population.

Microbiota-directed interventions are being investigated in the transplantation setting, particularly for gastrointestinal GVHD, but the evidence remains heterogeneous and these approaches are not established as standard therapy in allo-HCT recipients [32,33,34]. For example, Biernat et al. reported two patients with steroid-resistant intestinal acute GVHD treated with fecal microbiota transplantation (FMT), with differing clinical responses [32]. Broader transplantation literature similarly indicates that FMT remains an evolving intervention for which optimal patient selection, timing, preparation, endpoints and safety procedures have not been established [33,34].

MPN-specific interventional evidence is limited to isolated clinical observations. Yang et al. described a 69-year-old patient with primary myelofibrosis and recurrent Escherichia coli sepsis in whom the gastrointestinal tract was considered the probable infectious source [35]. After control of the acute infection, the patient underwent two administrations of washed microbiota transplantation (WMT) through a colonic transendoscopic enteral tube and had no further reported febrile episodes during the subsequent follow-up period [35]. Although directly relevant to an MPN patient, this single case does not establish efficacy of WMT for infection prevention or treatment in MPNs and should not be generalized beyond the reported clinical context.

Safety remains a major constraint for microbiota transplantation in immunocompromised patients. Donor-derived transmission of multidrug-resistant organisms has been documented; notably, ESBL-producing E. coli bacteremia occurred in two FMT recipients exposed to material from the same donor, with one fatal outcome [36]. These events emphasize the importance of rigorous donor screening, pathogen testing and standardized safety procedures [36,37]. Accordingly, FMT or WMT should not currently be considered as routine microbiota-directed therapy for MPNs. Their potential role in selected post-transplant, GVHD-related or infection-refractory settings remains investigational and distinct from evidence concerning the microbiota–inflammation relationship in classical MPN biology [31,32,33,34,35,36,37,38].

## 8. Supportive Care Context: Nutrition and Microbiota-Related Strategies in MPNs

Among supportive care approaches, nutrition has the most direct connection to microbiota-related research in MPNs. The NUTRIENT trial demonstrated that Mediterranean-style dietary counseling is feasible in patients with MPNs and can achieve good adherence, but over a 15-week intervention no significant changes in inflammatory cytokines or overall gut microbiota diversity and composition were observed [15,27]. These findings support dietary intervention as a feasible component of supportive care, but do not establish a microbiota-modifying, anti-inflammatory or disease-modifying effect in MPNs.

Broader integrative care reviews have discussed nutrition together with physical activity, mind–body interventions, supplements and other supportive approaches in MPNs [39,40]. However, these data are predominantly review-level, heterogeneous and frequently derived from small studies or from contexts not specifically designed to evaluate microbiota-related outcomes [39,40]. For the purposes of microbiota research in MPNs, nutrition therefore represents the most directly relevant supportive-care domain, whereas other integrative interventions should not be interpreted as evidence for microbiota modulation or MPN disease modification.

Evidence supporting probiotics, prebiotics, postbiotics or other microbiota-directed supplements specifically in MPNs is currently insufficient. Their biological plausibility should not be equated with demonstrated clinical benefit, and safety may be particularly relevant in patients with advanced disease, immunosuppression, active infection or post-transplant status. Accordingly, no nutrition-based or other supportive care intervention is currently validated as a microbiota-targeted treatment for MPNs, and such approaches should remain adjunctive to evidence-based hematologic care [33,34,36,37,39,40].

The principal MPN-specific human studies evaluating gut microbiota and microbiota-related molecular signals are summarized in Table 2. Contextual preclinical, causal inference, transplantation-related and supportive-care evidence is summarized separately in Supplementary Table S1.

## 9. Strengths and Limitations of This Review

This critical narrative review provides a structured synthesis of an emerging and heterogeneous body of literature by distinguishing MPN-specific human observations from mechanistic evidence derived primarily from preclinical or non-MPN settings, exploratory causal inference and multi-omics studies, and adjacent transplantation or supportive care evidence. This approach allows disease-specific findings to be interpreted separately from biological plausibility and translational context, without assigning formal grades of methodological quality or certainty.

Several limitations of the review methodology should be acknowledged. The literature search relied primarily on PubMed and was supplemented by targeted searches for mechanistic, methodological and translational context; consequently, relevant publications indexed exclusively in other databases may have been missed. Study identification, screening and initial evidence categorization were performed by one author, without duplicate independent screening or formal adjudication. In keeping with the critical narrative design, no PRISMA-based systematic selection process, quantitative meta-analysis, formal risk-of-bias assessment or evidence certainty grading was undertaken. These features introduce the possibility of selection and interpretative bias and limit the reproducibility achievable with a formal systematic review.

Limitations of the underlying evidence base are equally important. The number of independent MPN-specific human studies remains small, and several publications originate from the same investigative groups or from partially or substantially overlapping patient populations [12,13,14,15,22,23,27]. Most available studies are observational or cross-sectional and involve modest sample sizes, while populations differ in MPN subtype, driver mutation status, disease stage, age, treatment exposure, antibiotic use, diet, body mass index, infections and metabolic comorbidities. Microbiome studies also differ in sample collection, sequencing methodology, taxonomic resolution and analytical pipelines, limiting direct comparison across cohorts. Moreover, taxonomic differences do not by themselves demonstrate functional microbiome alteration, and functional validation through microbial pathways, metabolomics and host–microbial interactions remains limited. Mendelian randomization findings depend on genetic-instrument assumptions and should remain hypothesis generating, whereas microbial molecular signals detected in blood or bone marrow require particular caution because these are low-microbial-biomass compartments susceptible to contamination, batch effects and analytical variability [16,17,18,19,28,30]. Finally, much of the mechanistic and transplantation-related literature discussed in this review is derived from non-MPN settings and should not be interpreted as direct evidence of MPN pathogenesis.

## 10. Conclusions and Future Directions

Current MPN-specific human studies indicate that gut microbial composition and microbiota-related molecular signals are associated with inflammatory phenotype, driver mutation status, treatment exposure and dietary context [11,12,13,14,15,22,23,26,27]. Among these observations, the association between JAK2V617F-positive disease and differences in gut microbial composition is the most consistently reported disease-specific signal to date [12,13,14]. However, the available studies remain limited in number, several derive from overlapping or related patient populations, and most are observational or cross-sectional. Consequently, a reproducible and functionally defined MPN-associated dysbiosis profile has not been established, and taxonomic differences alone should not be interpreted as evidence of functional microbiome disruption.

Future MPN microbiome studies should prioritize multicenter, longitudinal designs with standardized preanalytical and analytical procedures, including sample collection, storage, DNA extraction, appropriate negative controls and harmonized bioinformatic pipelines [19]. Study populations should be characterized beyond broad MPN subtype and driver mutation status. Relevant variables include PV, ET, prefibrotic PMF and overt PMF classification; JAK2V617F allele burden; CALR mutation type; MPL and additional somatic mutations; disease stage and fibrosis grade; splenomegaly; previous thrombosis; treatment exposure; antibiotic use; diet; body mass index; infections; and metabolic comorbidities. Functional characterization should complement taxonomic profiling through microbial pathway analysis, metabolomics and integrated assessment of host inflammatory and molecular features. Such designs will be necessary to determine whether observed microbial differences have biological significance beyond association.

At present, there is no validated microbiota-based biomarker for MPNs, no validated microbial profile for diagnosis, risk stratification or disease monitoring, and insufficient evidence to recommend microbiota-targeted interventions as part of standard MPN treatment. Dietary intervention has demonstrated feasibility in patients with MPNs; however, the available short-term data do not demonstrate consistent microbiome remodelling or anti-inflammatory effects [15,27]. Microbiota transplantation remains investigational in hematologic and post-transplant settings and carries specific safety considerations in immunocompromised patients [31,32,33,34,35,36,37,38]. The principal contribution of current microbiota research in MPNs is therefore the identification of a biologically plausible and testable interface between the intestinal ecosystem, host inflammation, molecular disease architecture and hematopoietic biology. Establishing whether this interface has causal, prognostic or therapeutic relevance will require independent replication, functional validation and carefully standardized prospective studies.

## Figures and Tables

**Table 1:** Evidence categorization framework used for literature synthesis.

Evidence Domain	Type of Evidence Included	Relationship to MPN Evidence	Main Limitations	Interpretative and Translational Relevance
**MPN-specific human studies**	Studies evaluating fecal microbiota composition, inflammatory markers, driver mutation status, treatment exposure, extracellular vesicle-associated microbial signatures, or nutritional interventions in patients with PV, ET, PMF, prefibrotic PMF, or related MPN entities.	**Disease-specific human data**	Predominantly observational or cross-sectional designs; modest cohort sizes; heterogeneous MPN subtypes; variable treatment exposure; incomplete control for diet, antibiotics, and other confounders; and differences in sequencing methodology.	Provides the most disease-specific evidence currently available, but findings remain associative and require independent and longitudinal validation before use as biomarkers or for clinical decision-making.
**Mechanistic evidence from preclinical or non-MPN settings**	Studies addressing intestinal barrier dysfunction, microbial metabolites, low-grade endotoxemia, LPS/TLR4 signaling, NF-κB activation, thrombo-inflammation, hematopoietic stem cell regulation, and bone marrow microenvironmental remodeling.	**Indirect and hypothesis-generating evidence**	Mostly derived from preclinical models or non-MPN inflammatory, cardiovascular, or hematopoietic settings; biological plausibility does not establish an MPN-specific mechanism or causal relationship.	Provides biological plausibility for potential microbiota–inflammation–hematopoiesis interactions but should not be interpreted as demonstrating these pathways in patients with MPNs.
**Exploratory causal-inference and multi-omics studies**	Mendelian randomization and mediation analyses, circulating microbial signatures, bone marrow-derived microbial molecular signals, and integrated proteomic or metabolomic approaches.	**Exploratory evidence; directness varies by study design and population**	Dependent on methodological assumptions, population-specific datasets, taxonomic resolution, pleiotropy, analytical pipelines, and contamination or batch effects in low-microbial-biomass samples.	Generates candidate taxa, metabolites, and host–microbe pathways for replication and functional validation; does not establish clinical utility or biological causality.
**Adjacent hematologic and transplantation-related evidence**	Studies, case reports, or reviews addressing fecal or washed microbiota transplantation, GVHD, recurrent infections, antimicrobial resistance, and microbiota disruption in immunocompromised hematologic patients.	**Adjacent clinical evidence, generally not MPN-specific**	Frequently derived from case reports, case series, or allo-HCT populations rather than classical MPN cohorts; major differences in treatment exposure, immune status, and clinical context.	Provides safety and translational context for selected patients with advanced or post-transplant MPNs; microbiota transplantation remains investigational.
**Supportive-care and intervention-related evidence**	Studies and reviews addressing nutrition, symptom burden, quality of life, and microbiota-related supportive strategies in MPN care.	**MPN-specific for some supportive-care outcomes; exploratory for microbiota-specific effects**	Heterogeneous interventions, small samples, short follow-up, and limited evidence linking microbiota modification to clinical outcomes.	Supports investigation of nutrition and supportive-care interventions but does not establish microbiota-directed treatment or disease-modifying efficacy.

**Note:** The categories describe the clinical or biological context and directness of the available evidence and do not represent formal ratings of methodological quality or certainty. No formal risk-of-bias or evidence-certainty grading system was applied. **Abbreviations:** allo-HCT, allogeneic hematopoietic cell transplantation; ET, essential thrombocythemia; LPS, lipopolysaccharide; MPN, myeloproliferative neoplasm; NF-κB, nuclear factor kappa B; PMF, primary myelofibrosis; PV, polycythemia vera; TLR4, Toll-like receptor 4.

**Table 2:** MPN-specific human studies evaluating gut microbiota and microbiota-related molecular signals.

Study	Population and Sample Size	Study Design and Biological Material	Treatment Exposure	Main Findings	Major Limitations and Potential Confounders
**El Alaoui et al., 2018** [26]; **Oliver et al., 2022** [11]	25 patients with MPNs (11 PV, 8 ET, 6 MF) and 25 non-MPN controls.	Pilot observational study; triplicate fecal samples collected over 1 week; 16S rRNA gene sequencing; inflammatory cytokines assessed in a subset.	Heterogeneous MPN-directed therapy; treatment regimens were recorded but the study was not designed or powered for treatment-specific comparisons.	Global alpha diversity did not differ significantly between MPN and control groups. MPN status explained a small proportion of overall microbial compositional variance, while taxon-specific differences, including lower *Phascolarctobacterium*, and associations between selected microbial features and inflammatory cytokines were reported.	Small, cross-sectional cohort; marked interindividual variability; treatment heterogeneity; diet, lifestyle and cohabitation may influence microbial composition. The 2018 abstract and 2022 full report have the same sample size, study structure and overlapping investigators and should not be considered independent replications.
**Eickhardt-Dalbøge et al., 2023** [12]	54 patients with ET and 42 healthy controls; 36 ET patients were JAK2V617F-positive.	Cross-sectional observational study; fecal microbiota profiling using 16S rRNA gene sequencing.	IFN-α, n = 11; hydroxyurea, n = 23; no cytoreductive treatment, n = 15; other treatment, n = 5.	Microbial richness and composition differed between ET and healthy controls, with lower abundance of several Firmicutes-related taxa. Differences relative to controls were more pronounced among JAK2V617F-positive than JAK2V617F-negative ET patients.	Cross-sectional design; heterogeneous treatment exposure; relatively small molecular subgroups; differences in sex distribution and comorbidity burden between ET and controls; functional consequences of the taxonomic findings were not established.
**Eickhardt-Dalbøge et al., 2023** [14]	102 patients with PV and 42 healthy controls.	Cross-sectional observational study; fecal microbiota profiling using V3–V4 16S rRNA gene sequencing.	No cytoreductive therapy, n = 19; hydroxyurea, n = 37; IFN-α2, n = 23; IFN-α2 plus ruxolitinib, n = 23.	Patients with PV showed lower alpha diversity and lower relative abundance of several Firmicutes taxa than controls. Microbial composition also differed across treatment groups, with IFN-α2-treated patients showing profiles closer to those of healthy controls.	Cross-sectional design precludes attribution of microbial differences to treatment; treatment groups differed in age, treatment duration and clinical characteristics; residual dietary and environmental confounding remains possible.
**Eickhardt-Dalbøge et al., 2024** [13]	227 patients with MPNs: 54 ET, 114 PV, 13 prefibrotic PMF and 46 overt MF; 42 healthy controls. JAK2V617F, n = 177; CALR, n = 30.	Expanded cross-sectional cohort; fecal microbiota profiling using 16S rRNA gene sequencing; analyses by MPN subtype and driver mutation.	Mixed treatment exposure; patients with recent changes in cytoreductive therapy were excluded; antibiotic exposure within the preceding 2 months was an exclusion criterion.	MPN patients showed higher observed richness and lower abundance of several Firmicutes-related taxa, including *Faecalibacterium*. Microbial composition was more strongly associated with JAK2V617F status than with clinical MPN subtype; CALR-positive patients showed profiles more similar to healthy controls.	**Not an independent replication of** [12] **and** [14]: the cohort incorporated the previous ET and PV datasets and added 12 PV, 13 prefibrotic PMF and 46 MF patients. Cross-sectional design; heterogeneous treatment exposure; limited power for treatment-specific analyses in some subgroups; taxonomic findings do not establish functional dysbiosis.
**Avelar-Barragan et al., 2023** [27]; **Mendez Luque et al., 2024** [15]	31 patients with MPNs randomized; 28 constituted the final evaluable cohort (15 Mediterranean-diet counseling, 13 standard U.S. dietary counseling).	Randomized phase I pilot dietary study; 15-week follow-up; longitudinal stool and blood sampling; shotgun metagenomic microbiome analysis in the companion microbiome study.	Background MPN-directed treatment was permitted and heterogeneous; dietary counseling was the randomized intervention.	Mediterranean-style dietary counseling was feasible with good adherence. Overall gut microbiome diversity and composition remained relatively stable, and no significant changes in inflammatory cytokines were demonstrated; exploratory associations between microbial features, disease characteristics and inflammatory markers were reported.	Small pilot study; short duration; heterogeneous MPN subtype and background therapy; not powered for clinical, inflammatory or microbiome efficacy endpoints. [15] and [27] are complementary reports from the same NUTRIENT trial and are not independent cohorts.
**Barone et al., 2021** [22,23]	EV-PV cohort: 38 patients with PV; [22] additionally included 30 healthy donors.	Observational clinical-biological study; plasma-derived extracellular vesicles, EV-associated microbial DNA assessed by 16S rRNA sequencing; fecal microbiota evaluated in [22,23] assessed associations with thrombosis history and marrow fibrosis.	All PV patients had undergone phlebotomy and antiplatelet therapy; hydroxyurea was ongoing in 81.6%; no patients were receiving interferon or ruxolitinib.	EV-associated microbial DNA composition differed between patients with PV and healthy donors, whereas fecal microbiota did not clearly separate the groups [22]. Within the PV cohort, EV-associated host and microbial DNA features were associated with previous thrombosis and marrow fibrosis [23].	Same EV-PV study population underlies the related reports; observational and cross-sectional analyses; substantial hydroxyurea exposure; blood-derived EV material represents a low-microbial-biomass compartment susceptible to contamination and analytical variability; findings require independent technical and clinical validation.
**Zhang et al., 2024** [18]	13 untreated JAK2V617F-positive patients: 7 ET and 6 prefibrotic PMF.	Exploratory multi-omics study of FFPE bone marrow biopsies; 4D-DIA proteomics and 2bRAD-M sequencing of microbial DNA-derived features.	No previous hydroxyurea, interferon or other MPN-directed cytoreductive therapy; no antibiotics, probiotics, prebiotics or synbiotics during the preceding 3 months.	Proteomic and microbial DNA-derived features differed between ET and prefibrotic PMF. Selected proteins and bacterial genera showed discriminatory performance within the study cohort.	Very small cohort; no healthy control or independent validation cohort; low-microbial-biomass FFPE bone marrow material; ET and prefibrotic PMF groups differed substantially in JAK2V617F allele burden; reported discriminatory performance is internal and does not establish clinical diagnostic utility.

**Note:** Related or overlapping publications are presented together where appropriate to avoid overestimating the number of independent patient cohorts. The table summarizes MPN-specific human observations and does not constitute a formal grading of methodological quality or certainty. Taxonomic differences or microbial DNA-derived signals should not be interpreted as evidence of functional dysbiosis without complementary functional validation. No microbiota-based biomarker, risk-stratification profile or microbiota-targeted intervention has been clinically validated for MPNs. Abbreviations: ET—essential thrombocythemia; EV—extracellular vesicle; FFPE—formalin-fixed paraffin-embedded; IFN-α—interferon alpha; MF—myelofibrosis; MPN—myeloproliferative neoplasm; PMF—primary myelofibrosis; PV—polycythemia vera.

## Data Availability

No new data were created or analyzed in this study. Data sharing is not applicable to this article.

## Supplementary Materials

The following supporting information can be downloaded at https://www.mdpi.com/article/10.3390/biomedicines14092074/s1, Table S1: Contextual preclinical, causal-inference, multi-omics, transplantation related and supportive-care evidence.
