# Supplementary material for: Microbiota–Inflammation Crosstalk in Myeloproliferative Neoplasms: MPN-Specific Human Data, Mechanistic Plausibility and Translational Priorities
Source: Biomedicines. 2026 Sep 15;14(9):2074. doi: 10.3390/biomedicines14092074 (PMC13604808; doi:10.3390/biomedicines14092074)
Supplement: Supplementary file 1 [file biomedicines-14-02074-s001.zip › biomedicines-4505055-supplementary.docx]

**Supplementary material for:** Microbiota–Inflammation Crosstalk in Myeloproliferative Neoplasms: MPN-Specific Human Data, Mechanistic Plausibility and Translational Priorities

**Supplementary Table S1. Contextual preclinical, causal-inference, multi-omics, transplantation-related and supportive-care evidence**

**Note:** This table summarizes contextual or adjacent evidence used in the narrative synthesis and should not be interpreted as direct evidence of MPN pathogenesis or as a formal grading of methodological quality or certainty. Reference numbers correspond to the main manuscript. MPN-specific observational and clinical human studies are summarized separately in Table 2 of the main manuscript.

| **Study** | **Evidence context / design** | **Population or model and method** | **Main findings relevant to the review** | **Principal limitations / interpretative caveat** |
| --- | --- | --- | --- | --- |
| **Tadokoro et al., 2018 [24]** | Preclinical mechanistic study | Spred1-deficient murine model exposed to high-fat diet; hematopoietic and signaling analyses with assessment of microbiota-dependent effects. | High-fat diet in Spred1-deficient mice produced ERK hyperactivation, abnormal hematopoietic stem-cell self-renewal, severe anemia and an MPN-like phenotype; the hematopoietic abnormalities were partly mediated by alterations in the gut microbiota. | Murine model and not a model of classical human MPN. Supports biological plausibility only and does not establish that microbiota alterations initiate MPNs in patients. |
| **Josefsdottir et al., 2017 [25]** | Preclinical microbiota-depletion study | Mice treated with broad-spectrum antibiotics; germ-free comparison; hematopoietic phenotyping and fecal microbiota transfer. | Antibiotic-induced microbiota depletion was associated with anemia, thrombocytosis, leukopenia, depletion of hematopoietic stem and multipotent progenitor cells, and partial correction of hematopoietic abnormalities after fecal microbiota transfer. Germ-free mice showed similar abnormalities. | Not MPN-specific and represents profound experimental microbiota depletion. The findings support a role for intestinal microbiota in hematopoietic homeostasis but cannot be directly extrapolated to chronic MPN biology. |
| **Kan et al., 2025 [16]** | Exploratory two-sample Mendelian randomization, mediation and multivariable MR | GWAS-based analysis of 196 gut microbial taxa and 526 plasma metabolites in relation to MPN risk; microbial instruments derived from MiBioGen; MPN outcome data from large genetic datasets; external 16S dataset used for exploratory validation. | The authors reported MR associations involving seven microbial taxa and seventeen plasma metabolites. Mediation models linked the Eubacterium xylanophilum group to MPN through succinylcarnitine and lysine. | Dependent on genetic-instrument validity, population-specific GWAS data, taxonomic resolution and absence of horizontal pleiotropy. Genetically predicted microbial abundance is not equivalent to directly measured or clinically modifiable exposure; findings require independent functional validation. |
| **Chen et al., 2024 [28]** | Bidirectional Mendelian randomization across hematologic malignancies | GWAS-based gut microbial traits evaluated against several common hematologic malignancies, including MPNs, using forward and reverse MR analyses. | Within the MPN analysis, forward MR identified associations involving Coprococcus 3 and the Eubacterium hallii group; reverse MR also identified several associations between MPN susceptibility and microbial traits. | Broader hematologic-malignancy analysis rather than an MPN-specific functional study. Subject to MR assumptions, limited microbial taxonomic resolution, population dependence and multiple exploratory comparisons. |
| **Xu et al., 2020 [29]** | Host genetics–microbiome association study with bidirectional MR | Chinese population of 1,475 participants; microbiome GWAS, heritability analyses and bidirectional MR evaluating relationships between gut microbial traits and complex diseases. | Demonstrated host genetic influences on selected microbial traits and reported shared or distinct disease-associated microbial features; systemic lupus erythematosus and chronic myeloid leukemia shared some gut microbiome features. | Not focused on Philadelphia-negative MPNs. Provides only broad contextual support for host genetics–microbiome interactions and should not be interpreted as direct MPN evidence. |
| **Woerner et al., 2022 [17]** | Sequencing-based multi-disease myeloid malignancy study | Existing deep DNA-sequencing data from 1,870 patients with myeloid malignancies, including 354 with MPN; 1,756 bone marrow and 114 peripheral blood samples; bacterial, fungal and viral reads assessed after quality filtering. | Microbial DNA-derived patterns differed across myeloid disease subtypes and were associated with host mutations and blast percentage. Bacterial-content classifiers showed some discrimination among disease subtypes; MPN-related JAK2-mutant versus wild-type discrimination was limited. | Not restricted to MPNs. Blood and bone marrow are low-microbial-biomass compartments susceptible to contamination and batch effects. The study did not establish an MPN-specific prognostic microbial signature or a viable circulating/tissue-resident microbiome. |
| **van Lier et al., 2023 [31]** | Review / translational synthesis in allogeneic HCT | Review of intestinal microbiota disruption, graft-versus-host disease and transplant outcomes after allogeneic hematopoietic cell transplantation. | Summarizes associations between microbiome injury, loss of diversity, intestinal domination, immune dysregulation, GVHD and infectious complications after allo-HCT. | Adjacent transplantation evidence rather than evidence for classical MPN pathogenesis. Most relevant to selected patients with myelofibrosis or advanced MPN undergoing allo-HCT. |
| **Biernat et al., 2020 [32]** | Two case reports and literature review | Two allo-HCT recipients with steroid-resistant intestinal acute GVHD treated with fecal microbiota transplantation. | Clinical responses differed between patients; the report illustrates the potential feasibility of FMT in selected steroid-resistant intestinal GVHD settings. | Very small case-based evidence with heterogeneous response. Not MPN-specific and insufficient to establish efficacy or routine clinical use. |
| **Zlitni et al., 2020 [38]** | Longitudinal strain-resolved metagenomic study | Single patient undergoing hematopoietic cell transplantation; read-cloud sequencing used for high-resolution tracking of stool microbial strains and mobile genetic elements. | Demonstrated marked microbiota disruption during intensive treatment and strain-level dynamics involving Bacteroides caccae and mobile elements associated with antimicrobial resistance. | Single-patient transplantation context and not MPN-specific. Useful as a methodological illustration of ecological and resistance dynamics rather than evidence for MPN biology. |
| **Yang et al., 2024 [35]** | MPN-specific case report of washed microbiota transplantation | 69-year-old patient with primary myelofibrosis and recurrent Escherichia coli sepsis; WMT administered through a transendoscopic colonic tube after control of the acute infection. | No further recurrent febrile episodes were reported during subsequent follow-up after WMT in the published case. | Single-patient observation without a comparator; cannot establish efficacy, causality or generalizability. WMT remains investigational in this context. |
| **Gray and DeFilipp, 2023 [33]** | Review / expert perspective | Review of fecal microbiota transplantation for acute GVHD after allogeneic HCT, including discussion of extension into pediatric populations. | Summarizes early clinical experience and key development questions for FMT in acute GVHD. | Adjacent allo-HCT literature rather than direct MPN evidence. Clinical efficacy, patient selection, timing and safety remain incompletely defined. |
| **Habibi and Rashidi, 2023 [34]** | Review / translational synthesis | Review of FMT studies in hematopoietic cell transplant and cellular-therapy recipients. | Highlights lessons from early FMT experience, including donor selection, product preparation, timing, endpoints, engraftment assessment and safety considerations. | Review-level adjacent evidence. The intervention remains investigational in many hematologic and transplant settings and should not be generalized to routine MPN care. |
| **Ng et al., 2024 [37]** | Systematic review of FMT donor-screening protocols | Review of donor-screening recommendations and gaps relevant to fecal microbiota transplantation. | Emphasizes rigorous donor assessment, pathogen testing and surveillance as essential components of FMT safety. | Safety and methodological context rather than MPN-specific evidence; screening recommendations may evolve with emerging pathogens and regulatory requirements. |
| **DeFilipp et al., 2019 [36]** | Clinical safety report / donor-derived transmission event | Immunocompromised recipients exposed to FMT material from the same donor; genomic analysis used to investigate transmission. | ESBL-producing Escherichia coli bacteremia occurred in two recipients and was linked to the donor material; one recipient died. | Safety-focused report rather than efficacy evidence. Demonstrates the potential for serious donor-derived infection and supports stringent screening in immunocompromised patients. |
| **Singh et al., 2024 [39]** | Narrative review of integrative oncology in MPNs | Review of nutrition, physical activity, mind–body approaches, supplements and other supportive-care strategies for symptom burden and quality of life in MPNs. | Provides a broader supportive-care framework and identifies nutrition as one potential interface with inflammation and microbiota-related research. | Heterogeneous, predominantly review-level evidence; many interventions are supported by small studies or extrapolation and do not demonstrate microbiota modification or disease modification. |
| **Andreazzoli et al., 2025 [40]** | Narrative review of integrative MPN management | Review of complementary and integrative approaches including nutrition, microbiota-related strategies, supplements, mind–body techniques and acupuncture. | Discusses supportive approaches as adjuncts to conventional MPN therapy and emphasizes safety and individualized clinical guidance. | Review-level evidence and not proof of microbiota-mediated benefit or disease modification. These approaches should remain adjunctive to evidence-based hematologic care. |

**Abbreviations:** allo-HCT, allogeneic hematopoietic cell transplantation; FMT, fecal microbiota transplantation; GVHD, graft-versus-host disease; HCT, hematopoietic cell transplantation; HSC, hematopoietic stem cell; MPN, myeloproliferative neoplasm; MR, Mendelian randomization; WMT, washed microbiota transplantation.
